# Decoding Intracranial EEG With Machine Learning: A Systematic Review

**DOI:** 10.3389/fnhum.2022.913777

**Published:** 2022-06-27

**Authors:** Nykan Mirchi, Nebras M. Warsi, Frederick Zhang, Simeon M. Wong, Hrishikesh Suresh, Karim Mithani, Lauren Erdman, George M. Ibrahim

**Affiliations:** ^1^Faculty of Medicine, University of Toronto, Toronto, ON, Canada; ^2^Division of Neurosurgery, Hospital for Sick Children, Department of Surgery, University of Toronto, Toronto, ON, Canada; ^3^Institute of Biomedical Engineering, University of Toronto, Toronto, ON, Canada; ^4^Faculty of Medicine, University of Ottawa, Ottawa, ON, Canada; ^5^Program in Neuroscience and Mental Health, Hospital for Sick Children Research Institute, Toronto, ON, Canada; ^6^Vector Institute for Artificial Intelligence, MaRS Centre, Toronto, ON, Canada; ^7^Department of Computer Science, University of Toronto, Toronto, ON, Canada; ^8^Hospital for Sick Children, Toronto, ON, Canada; ^9^Institute of Medical Science, University of Toronto, Toronto, ON, Canada

**Keywords:** intracranial EEG (iEEG), seizure, epilepsy, neurorecording, machine learning, artificial intelligence, deep learning

## Abstract

Advances in intracranial electroencephalography (iEEG) and neurophysiology have enabled the study of previously inaccessible brain regions with high fidelity temporal and spatial resolution. Studies of iEEG have revealed a rich neural code subserving healthy brain function and which fails in disease states. Machine learning (ML), a form of artificial intelligence, is a modern tool that may be able to better decode complex neural signals and enhance interpretation of these data. To date, a number of publications have applied ML to iEEG, but clinician awareness of these techniques and their relevance to neurosurgery, has been limited. The present work presents a review of existing applications of ML techniques in iEEG data, discusses the relative merits and limitations of the various approaches, and examines potential avenues for clinical translation in neurosurgery. One-hundred-seven articles examining artificial intelligence applications to iEEG were identified from 3 databases. Clinical applications of ML from these articles were categorized into 4 domains: i) seizure analysis, ii) motor tasks, iii) cognitive assessment, and iv) sleep staging. The review revealed that supervised algorithms were most commonly used across studies and often leveraged publicly available timeseries datasets. We conclude with recommendations for future work and potential clinical applications.

## Introduction

Intracranial electroencephalography (iEEG) provides exquisite detail with which to study neural activity. Stereotactic EEG (sEEG), strips, grids and depth electrodes are among the most common iEEG modalities. Modern diagnostic and treatment strategies for neurological conditions such as epilepsy rely heavily on interpreting iEEG signals to extract meaningful information that can impact clinical decision making (Lachaux et al., 2003). Furthermore, the study of these signals can serve to uncover the neural syntax associated with both healthy and diseased states by using explainable ML algorithms to shed light on important features that differentiate these states.

Machine learning (ML) is a rapidly growing field that has shown significant potential in complex biomedical applications (Rajkomar et al., 2019). Simply, ML refers to algorithms that can make seemingly intelligent decisions after identifying and learning from hidden patterns in large pre-existing data. Training these algorithms on large biologic datasets for example has enabled the identification of complex patterns not apparent to human experts and informed intelligent decision-making without explicit programming (Kotsiantis et al., 2007). Examples of success in these endeavors include automated diagnostic algorithms in radiology (Syeda-Mahmood, 2018) and pathology (Bera et al., 2019).

**Figure 1:**
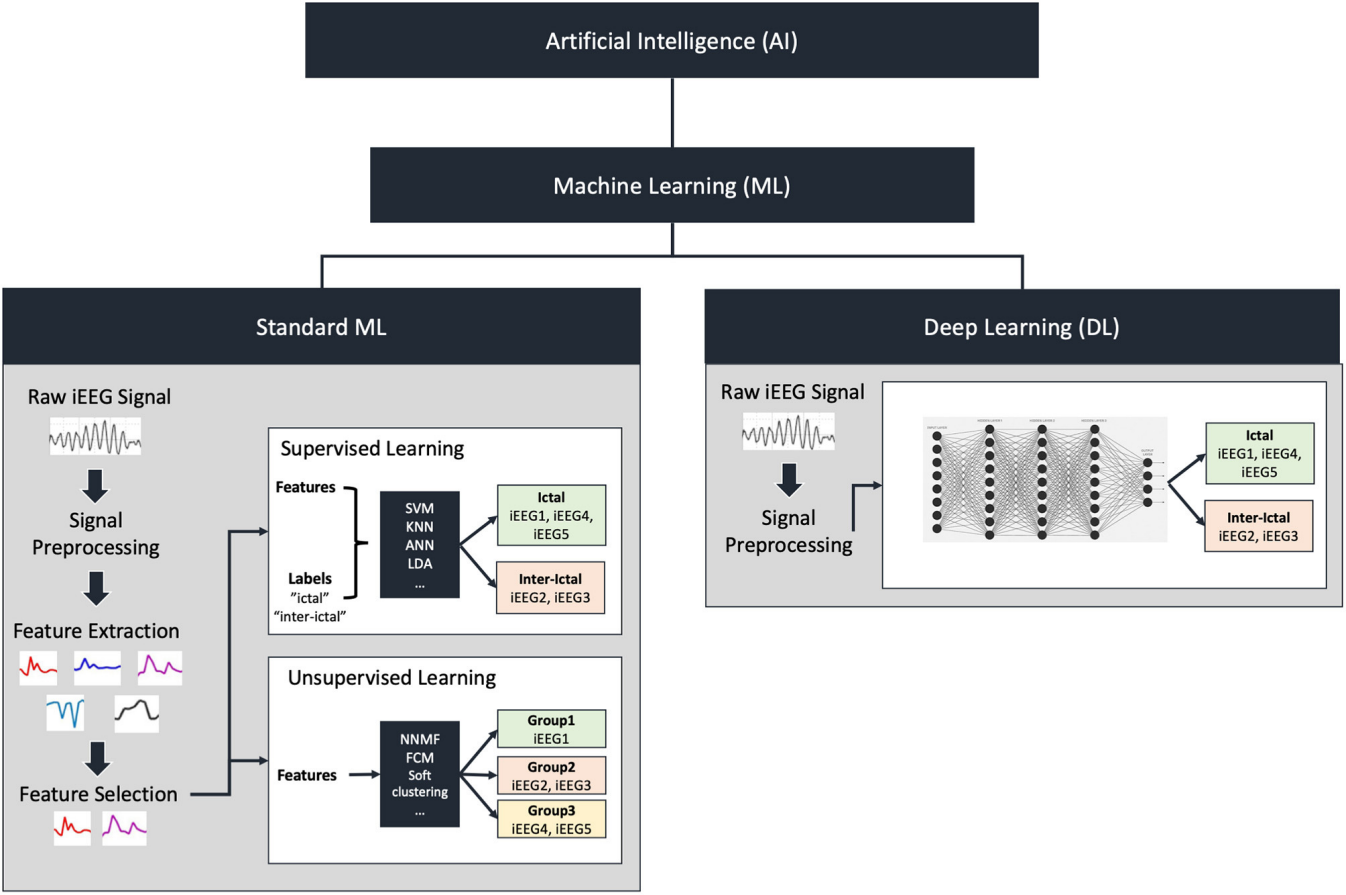
Overview of artificial intelligence and machine learning. Artificial intelligence includes machine learning (ML) which can be divided into standard machine learning or deep learning. In standard ML, raw iEEG signal is preprocessed followed by feature extraction and selection. Feature extraction involves breaking down the raw signal into individual quantifiable components such as power at various frequencies. Feature selection on the other hand involves selecting a subset of these components to be used to train a model. These features can either be fed to a supervised learning algorithm in addition to group labels, or an unsupervised learning algorithm that does not contain labels. Examples of supervised algorithms include support vector machines (SVM), K-nearest neighbors (KNN), artificial neural networks (ANN) or linear discriminant analysis (LDA). These classify the features into the group labels provided as inputs. Examples of unsupervised algorithms include non-negative matrix factorization (NNMF), fuzzy-c-means (FCM), and soft clustering. These will classify the features into groups based on similarity. Deep learning does not require feature extraction and selection. The processed or unprocessed signal can be fed directly into the deep learning model to classify the signals.

ML algorithms can be dichotomized into “supervised” and “unsupervised” learning approaches. An overview of these methodologies is illustrated in Figure 1. Supervised learning involves the use of labeled data during training such that the algorithm learns to associate certain patterns with a predefined label (for example, data associated with seizure vs. non-seizure states) (Kotsiantis et al., 2007). Unsupervised learning involves unlabelled data where the algorithm is provided a large dataset and learns to group certain signals together *without* a priori knowledge of classification. Each method possesses respective advantages and pitfalls. Deep learning approaches, which were initially designed based on a simplified model of interconnected nodes that mimic neuronal connections, also warrant special note. Each node is analogous to a cell body and the connections (“synapses”) between nodes are assigned weights during training analogous to the strength of synaptic connections between neurons. A primary advantage of deep learning compared to standard ML is that these algorithms can learn from the raw data and automatically extract meaningful information thereby bypassing human-intensive feature selection steps. However, it is important to consider that deep networks can take significantly longer to train and often require larger sample sizes than commonly available in neurosurgical populations.

Despite the potential for application of ML to iEEG data, evidence to support the utilization of different ML techniques is limited, and considerable heterogeneity has been reported in the literature. As such, the present systematic review aims to: 1) assess the current landscape of ML utilization in iEEG and 2) examine the relative benefits and limitations of ML approaches as they relate to specific neurosurgical problems.

## Methods

### Search Strategy

In order to identify published, peer-reviewed articles that employ ML in the context of iEEG data, we performed a systematic search of articles using 3 commonly accessed medicine and engineering databases: OVID MEDLINE, IEEE, and Web of Science. We selected these databases as we aimed to capture the breadth of applications of this technology in the fields of science, medicine, and engineering. The search was conducted on August 18^th^, 2020. Our inclusion criteria comprised of two categories of keywords which must appear in either the article title, abstract or article keywords. The first category comprised of machine learning keywords (“machine learning”, “deep learning”, “artificial intelligence”, “neural network$”), while the second category comprised of keywords relevant to intracranial EEG (“stereoelectroencephalography”, “stereotactic EEG”, “sEEG”, “electrocorticography”, “ECoG”, “intracranial EEG”, “intracranial electroencephalography”, “iEEG”). Relevant articles were required to contain at least one keyword from the first category and at least one from the second category. A detailed summary of our search strategy and yield is provided in the Supplementary Material 1.

### Article Filtering

**Figure 2:**
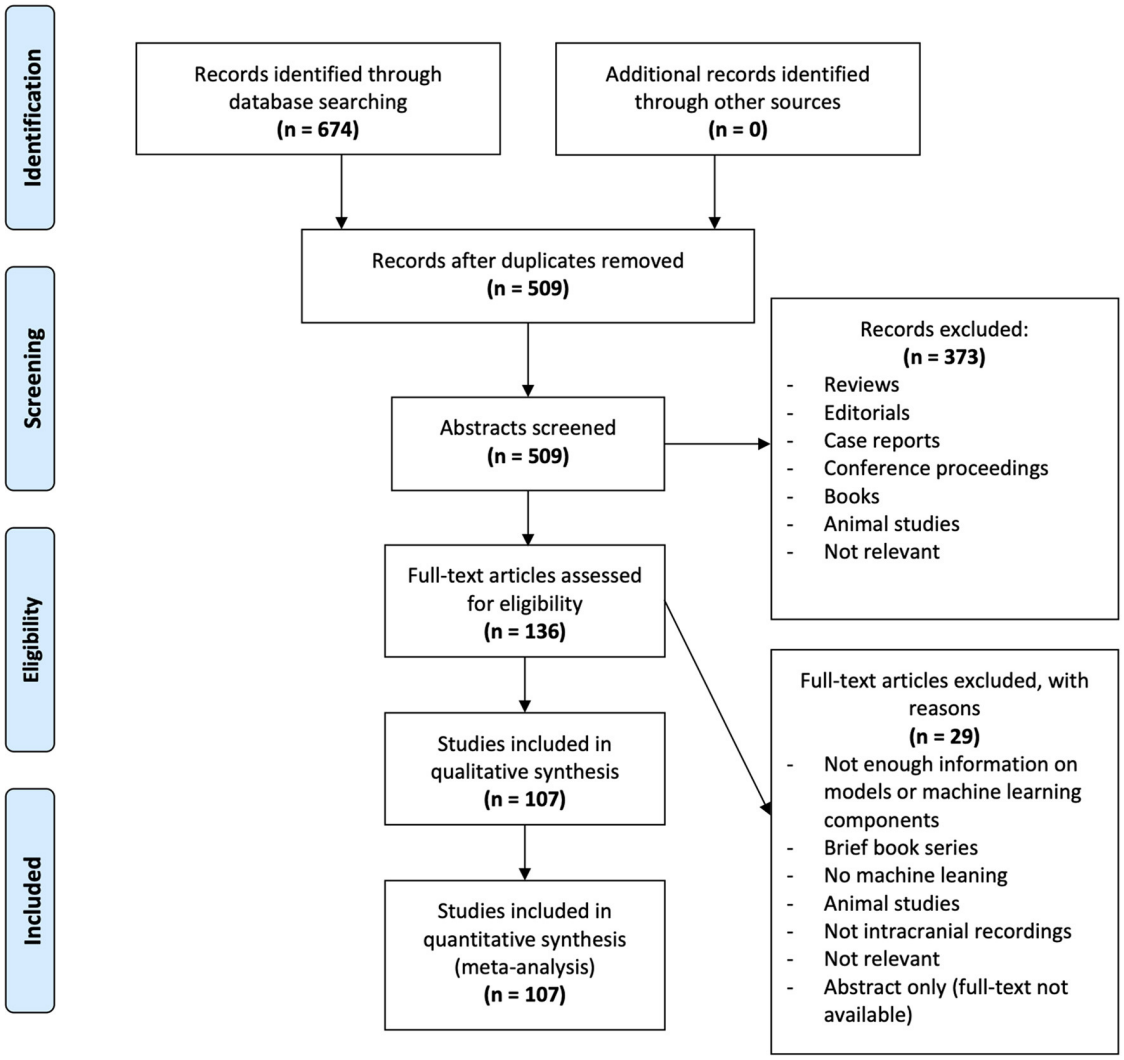
PRISMA analysis of articles searched, filtered and included in the systematic review. Six-hundred-seventy-four articles were identified through database searching in OVID MEDLINE, IEEE, and Web of Science. Duplicates were removed leaving 509 abstracts for screening. Three-hundred-seventy-three articles were excluded as they did not fit out inclusion criteria. From the remaining 136 articles, 29 were excluded following full-text screening. The remaining 107 articles were analyzed in this study.

The PRISMA guidelines (Moher et al., 2009) were followed to filter through articles in a systematic and transparent manner (Figure 2). Following our database search, 674 articles were found (OVID MEDLINE: 159, IEEE: 225, Web of Science: 290). One-hundred-sixty-five duplicates were removed leaving 509 for title and abstract filtering. Three-hundred-seventy-three articles were excluded following this first step of filtering as they did not fit our inclusion criteria for the following reasons: reviews, editorials, case reports, conference proceedings, book sections, animal studies, not relevant to intracranial EEG or machine learning. Two independent reviewers (NM, NW) then filtered the full text of 136 remaining articles. Disagreements were resolved by discussion and, when required, a third adjudicator (GMI). Twenty-nine articles were excluded for the following reasons: preliminary results, not clinical application, brief book series, not machine learning, animal studies, not intracranial EEG, not relevant, abstract only. Following this step, 107 articles were selected for this review.

### Data Collection

Each full-text article was reviewed and data regarding demographics, data acquisition, machine learning application, and types of features were gathered into a spreadsheet.

#### Demographics

Demographics data was composed of year of publication, country of origin, name of the journal, journal category (medical, engineering/computer science, or multidisciplinary). The country of origin was defined by the affiliation of the first author.

#### Data Acquisition

Data acquisition comprised of intracranial recording method. This includes sEEG, and electrocorticographic (ECoG) strips or grids, or depth electrodes. A fourth option was available if the studies did not specify the type of recording method used. The source of EEG data was also compiled as free-text entries to track whether studies used their own data, or data obtained from online databases.

#### Machine Learning

The application of machine learning algorithms was classified into four broad categories. Due to the heterogeneity of studies in these fields, studies in the seizure and motor categories were further subdivided as follows:

1. **Seizure**: seizure prediction/detection, surgical outcome prediction, pathological tissue detection, high-frequency oscillation (HFO) detection/classification, seizure onset zone (SOZ)/epileptic focus localization, bad channel detection, spike detection, tumor tissue detection.
2. **Motor**: movement classification, motor imagery, speech production.
3. **Cognitive tasks**
4. **Sleep Staging**.

The type of ML algorithm was categorized into four groups: Standard supervised ML includes popular algorithms such as support vector machines (SVM), K-nearest neighbors (KNN), single-layered artificial neural networks (ANN), linear discriminant analysis (LDA) and any others that did not fit the definition of deep learning. Deep learning algorithms include recurrent neural networks (RNN), convolutional neural networks (CNN) and multi-layered neural networks (LeCun et al., 2015). The third option applied to studies that employed both standard ML and deep learning methods. The last option included unsupervised machine learning algorithms such as non-negative matrix factorization (NNMF) amongst others. We also recorded whether a study employed one or multiple types of algorithm for purposes of comparing classification performance. Studies which compared their algorithm performance with previously published algorithms trained on the same data were considered one algorithm as they did not themselves train multiple algorithms.

A summary of relevant feature selection steps was also collected in order to understand the data extraction approach utilized by each study. For purposes of comparison, feature selection approaches were split into two main categories: 1) higher order feature selection algorithms; and 2) physically interpretable features (raw or hand-crafted). Studies that used higher-order feature-selection algorithms were defined as those in which the feature selection/pre-processing workflows contained one or more of: i) non-standard time-frequency transforms; ii) dimensionality reduction of feature space; iii) abstract feature selection algorithms; or iv) abstract features (entropy, fractal dimension, etc.).

Performance metrics of the best final algorithms from each paper were recorded to gain insight on relative performance based on algorithmic and feature selection decisions. As algorithm performance can only be fairly compared between studies where both studies use the same dataset, this step was only done in studies employing the same publicly available datasets further discussed in Section Intracranial EEG Datasets. Although accuracy may be considered a sufficient measure of performance, it holds many caveats whereby it may give a false impression of performance in highly unbalanced data. As such, we opted to record all performance metrics presented within each study to provide a more objective picture. These include accuracy, area-under-ROC-curve, sensitivity, specificity, precision, positive predictive value, and/or negative predictive value. As model specificity was most commonly reported in all included studies, this metric is used to compare performance of algorithms between studies. If this metric was not provided, accuracy is used for comparison. Where studies solely reported cross-validation performance, a single mean performance metric was tabulated. In others that split their data into validation and testing sets, both the validation and testing performance were compiled and the testing performance was used for comparison.

## Results

One-hundred-seven articles were included in the systematic review (Hellmann, 1999; Petrosian et al., 2000; D'Alessandro et al., 2005; Hill et al., 2006; Firpi et al., 2007a,b; Chan et al., 2008; Shenoy et al., 2008; Demirer et al., 2009; Liu et al., 2009, 2012; Mirowski et al., 2009; Scherer et al., 2009; Yanagisawa et al., 2009; Ayala et al., 2011; Chua et al., 2011; Kharbouch et al., 2011; Benz et al., 2012; Yang et al., 2012; Ikeda et al., 2014; McMullen et al., 2014; Zhang et al., 2014, 2015; Combrisson and Jerbi, 2015; Li et al., 2015; Memarian et al., 2015; Wang and Lyu, 2015; Yuan et al., 2015; Zheng et al., 2015; Boussen et al., 2016; Geng et al., 2016, 2020; Schrouff et al., 2016; Song and Zhang, 2016; Zhang and Parhi, 2016; Andrade et al., 2017; Antoniades et al., 2017; Chen et al., 2017; Combrisson et al., 2017; Elahian et al., 2017; Hosseini et al., 2017, 2018, 2020; Jrad et al., 2017; Khambhati et al., 2017; Kragel et al., 2017; Kremen et al., 2017, 2019; Parvez and Paul, 2017; Raghu and Sriraam, 2017; Sathish et al., 2017; Tomlinson et al., 2017; Vidyaratne and Iftekharuddin, 2017; Alickovic et al., 2018; Arora et al., 2018; Baud et al., 2018; Derner et al., 2018; Grinenko et al., 2018; Hermiz et al., 2018; Kiral-Kornek et al., 2018; Manzouri et al., 2018; Muller et al., 2018; O'Leary et al., 2018; Pan et al., 2018; Ramsey et al., 2018; Rutigliano et al., 2018; Shoaran et al., 2018; Truong et al., 2018, 2019; Tuyisenge et al., 2018; Varatharajah et al., 2018; Wu et al., 2018; Xie et al., 2018; Angrick et al., 2019a,b; Cimbalnik et al., 2019; Klimes et al., 2019; Lai et al., 2019, 2020; Livezey et al., 2019; Mahmoodian et al., 2019; Medvedev et al., 2019; Meisel and Bailey, 2019; Nejedly et al., 2019a,b; Pailla et al., 2019; Principe et al., 2019; Saboo et al., 2019; Sumsky and Santaniello, 2019; Thomas et al., 2019; Weidemann et al., 2019; Abou Jaoude et al., 2020; Akter et al., 2020; Burrello et al., 2020; Daoud and Bayoumi, 2020; Ghoroghchian et al., 2020; Gong et al., 2020; Karthick et al., 2020; Lian et al., 2020; Makaram et al., 2020; Makin et al., 2020; Rashid et al., 2020; RaviPrakash et al., 2020; Sciaraffa et al., 2020; Yu et al., 2020; Zhao et al., 2020; Zhu et al., 2020). Information regarding each article was collected into four categories: demographics, data acquisition, machine learning model, types of features. A succinct summary of studies employing publicly available datasets and reporting best AI model performance is presented in Table 1 while a more extensive table containing all studies is available in Supplementary Material 2.

**Table 1:** Summary of literature on artificial intelligence in intracranial EEG.

**Application**		**Dataset**	**Authors**	**Recording Method**	**Machine Learning**		**Features**
					**Category**	**Best Al Performance**	
Seizure	Seizure Detection / Prediction	Freiburg	Yu et al., 2020	Not specified	Standard	87.7% (ss)	Higher order
			Geng et al., 2020	Not specified	Deep	98.09% (ss), 98.69% (sp)	Higher order
			Lian et al., 2020	Not specified	Standard and Deep	95.67% (acc)	Higher order
			Truong et al., 2019	Not specified	Standard and Deep	88.86 (auc)	Higher order
			Meisel and Bailey, 2019	Strips/grids	Standard and Deep	Not reported	Higher order
			Mahmoodian et al., 2019	Not specified	Standard	96.8% (acc)	Higher order
			Alickovic et al., 2018	Not specified	Standard	100% (acc)	Higher order
			Parvez and Paul, 2017	Not specified	Standard	95.4% (acc)	Standard
			Zhang and Parhi, 2016	Not specified	Standard	100% (ss)	Standard
			Geng et al., 2016	Not specified	Standard	96.72% (ss)	Standard
			Song and Zhang, 2016	Not specified	Standard	85.73% (acc)	Higher order
			Zheng et al., 2015	Not specified	Standard	92% (ss)	Higher order
			Wang and Lyu, 2015	Not specified	Standard	98.8% (ss)	Higher order
			Yuan et al., 2015	Not specified	Standard	94.41% (ss), 96.97% (sp), 96.87% (acc)	Higher order
			Zhang et al., 2015	Not specified	Standard	92.94% (ss), 97.47% (sp), 97.57% (acc)	Higher order
			Zhang et al., 2014	Not specified	Standard	89.33% (ss)	Higher order
			Liu et al., 2012	Not specified	Standard	94.46% (ss), 95.26% (sp), 95.33 (acc)	Standard
			Chua et al., 2011	Not specified	Standard	78% (ss)	Standard
			Liu et al., 2009	Strips/grids and Depth electrodes	Deep	93.75% (ss)	Standard
			Mirowski et al., 2009	Strips/grids and Depth electrodes	Standard and Deep	71% (ss)	Higher order
		Bonn	Gong et al., 2020	Not specified	Standard and Deep	99.79% (acc CE), 98.96% (acc DE), 83.13% (acc CD), 98.75% (acc CD-E), 85.75% (acc CDE)	Standard
			Vidyaratne and Iftekharuddin, 2017	Not specified	Standard	99.8% (acc CD-E), 99% (ss CD-E), 100% (sp CD-E)	Higher order
			Raghu and Sriraam, 2017	Strips/grids and Depth electrodes	Standard	97.68% (acc CE), 94.56% (acc DE), 84.58% (acc CDE), 57.8% (acc CD)	Higher order
		EPILEPSIAE	Ghoroghchian et al., 2020	Not specified	Standard	0.89 (auc)	Standard
			Manzouri et al., 2018	Strips/grids and Depth electrodes	Standard	0.98 (auc)	Higher order
			O'Leary et al., 2018	Not specified	Standard	97.7% (ss)	Higher order
		Mayo-UPenn	Hosseini et al., 2018	Strips/grids and Depth electrodes	Standard	97% (acc), 98% (ss), 96% (sp)	Higher order
			Hosseini et al., 2017	Strips/grids	Standard and Deep	96% (acc), 97% (ss)	Higher order
		Bern-Barcelona	Sathish et al., 2017	Not specified	Standard	99.6% (acc)	Higher order
		Freiburg and Mayo-Upenn	Truong et al., 2018	Not specified	Deep	94.7% (acc; Freiburg), 96.18% (acc; M-UP cross-validation), 88.81% (acc; M-UP testing)	Standard
	SOZ / Epileptic Focus Localization	Bonn and Bern-Barcelona	Daoud and Bayoumi, 2020	Not specified	Deep	Bern-Barcelona 93.21% (acc), 90.50% (ss), 95.92% (sp) Bonn 96% (acc), 93% (ss), 99% (sp)	Standard
			Chen et al., 2017	Not specified	Standard	Bern-Barcelona 83.07% (acc), 83.05% (ss), 83.09% (sp) Bonn 88.00% (acc), 92.24% (ss), 83.76% (sp)	Higher order
		Mayo-Upenn	Hosseini et al., 2020	Depth electrodes	Standard and Deep	98% (acc), 96 (ss), 97% (sp)	Standard
Motor	Motor Imagery	BCI Competition III	Rashid et al., 2020	Strips/grids	Deep	Training 99.64% (acc), 100% (ss), 99.28 (sp) Testing 97% (acc), 96% (ss), 98% (sp)	Standard
			Li et al., 2015	Strips/grids	Standard	92% (acc)	Higher order
			Yang et al., 2012	Strips/grids	Standard	Training 88% (acc) Validation 80% (acc) Testing 80% (acc) Overall 86%	Higher order
			Demirer et al., 2009	Strips/grids	Standard	Training 95% Testing 73%	Higher order

***Keywords***

*Performance measures*

*acc, accuracy; auc, area under curve; sp, specificity; ss, sensitivity*.

### Demographics

**Figure 3:**
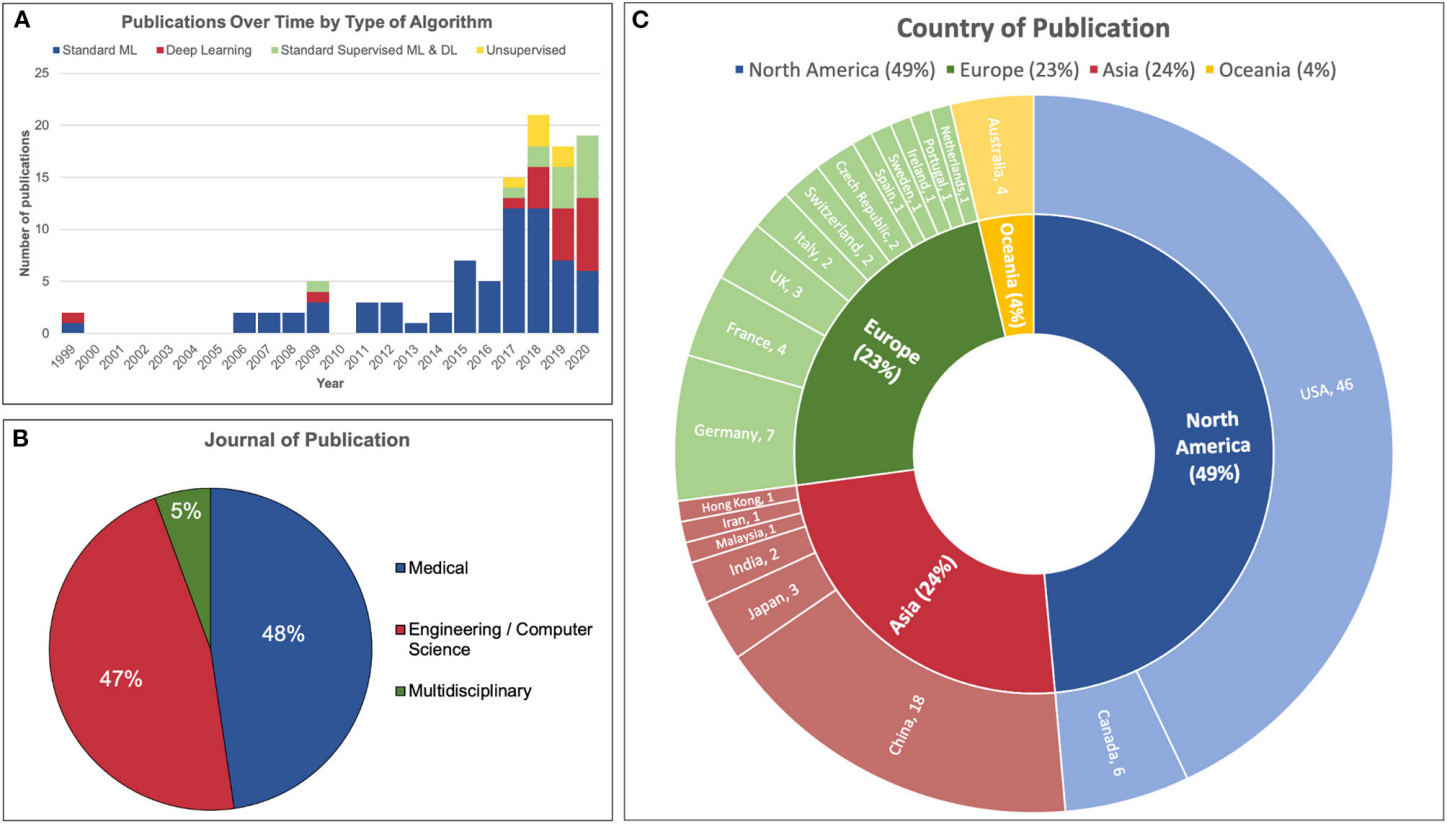
Demographics of 107 articles reviewed. **(A)** The number of publications in the field of machine learning and iEEG dramatically increased in 2017 incorporating more deep learning and unsupervised learning models. **(B)** Just under half of articles reviewed were published in a medical journal and a similar proportion were published in an engineering or computer science journal. A minority was published in a multidisciplinary journal. **(C)** Almost half of the studies were conducted in North America. The United States published the most papers, followed by China, Germany and Canada.

The number of publications per year is illustrated in Figure 3A. A dramatic increase in the number of publications was observed from 2017 onwards. The majority of articles before 2017 utilized standard machine learning algorithms as opposed to deep learning or unsupervised learning. For comparison, the use of deep learning or both (standard ML and deep learning) made up only 20% of articles published in 2017 compared to 68% of those published in 2020.

Fifty-one articles were published in medical journals while 50 were published in engineering or computer science journals. Six articles were published in multidisciplinary journals. This distribution is illustrated in Figure 3B.

The distribution of country of origin for each article is displayed in Figure 3C. The majority of articles originated from the United States of America (46/107, 42%), followed by China (18/107, 17%), Germany (7/107, 7%) and Canada (6/107, 6%).

### Data Acquisition

**Figure 4:**
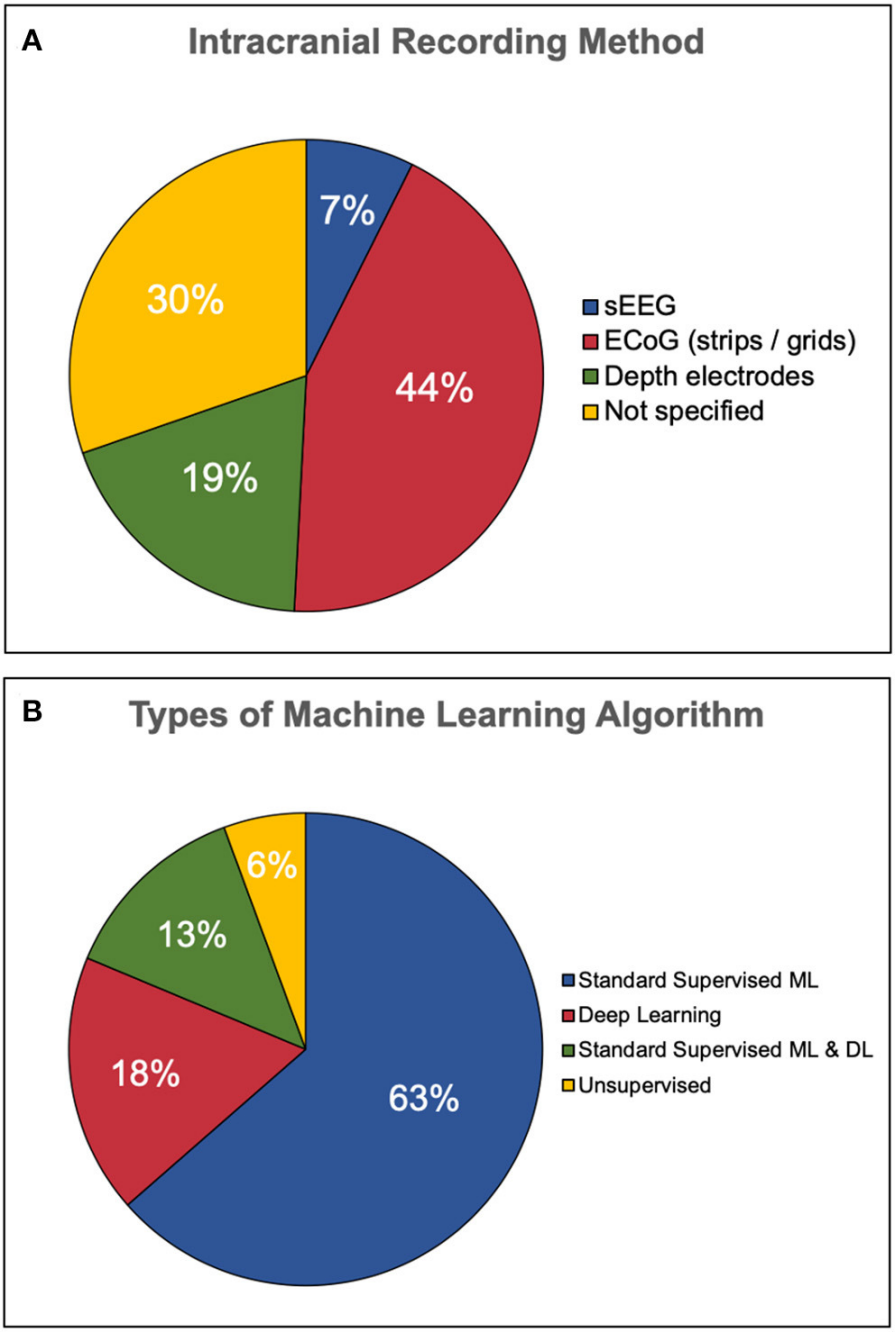
Types of iEEG recording and classification algorithm employed. **(A)** Less than half of the publications employed electrocorticography techniques such as strips and grids. The second most popular recording method was depth electrodes followed by a minority of stereotactic EEG. One third of the articles did not specify the type of recording method. **(B)** The majority of studies employ standard supervised machine learning (ML) only, while only 18% used deep learning (DL) only. Thirteen percent used both standard and deep learning while a minority used unsupervised learning.

The types of intracranial recording varied across each study. Some articles employed only one type of intracranial recording, while others used two methods. Articles which did not specify their method of intracranial EEG recording were simply labeled as unspecified iEEG. The distribution of intracranial recording method is illustrated in Figure 4A. Of those which specified a recording technique, ECoG strips or grids were used most frequently (53/107 articles), followed by depth electrodes (23/107) and sEEG (9/107). Thirty-seven articles were labeled as unspecified iEEG. Some articles used more than one method, hence this sum is greater than the number of articles.

Just over half of the articles analyzed in this review reported using their own datasets (57/107, 53%). The 50 remaining reported the use of at least one pre-existing dataset, either from their own research group or a freely available dataset available online. Some publications use more than one source of data.

#### Intracranial EEG Datasets

Pre-existing iEEG datasets are utilized in a number of these papers. The most popular of these include the Freiburg (Andrzejak et al., 2012) (21/50, 42%), the UBonn (Andrzejak et al., 2001) dataset (6/50, 12%), BCI Competition III (Blankertz et al., 2006) (4/50, 8%), Mayo Clinic (Stead et al., 2010) (3/50, 6%), Bern-Barcelona (3/50, 6%), and EPILEPSIAE (3/50, 6%). The Freiburg EEG database is composed of grid, strip and depth electrode recordings from 21 patients suffering from medically intractable focal epilepsy. The UBonn dataset is composed of strips and depth electrodes recorded from epilepsy patients. It is divided into 5 sets where A and B are scalp EEG and C, D and E are iEEG recordings from patients with temporal lobe epilepsy. Sets C and D are composed of inter-ictal intervals while set E shows ictal activity. The BCI Competition III includes 8 scalp and intracranial EEG datasets involving motor imagery and P300 speller paradigms. Specifically, only dataset 1 includes ECoG grids, while the remaining solely include scalp EEG. The Mayo Clinic dataset is composed of strips, grids and depth electrode recordings from 11 epilepsy patients. The Bern-Barcelona dataset comprises of strips and depth electrodes from 5 pharmacoresistant temporal lobe patients. Finally, the EPILEPSIAE database is the product of a collaboration between epilepsy centers in Portugal, France and Germany. It contains both scalp and intracranial EEG recordings including grids and depth electrodes.

### Machine Learning

**Figure 5:**
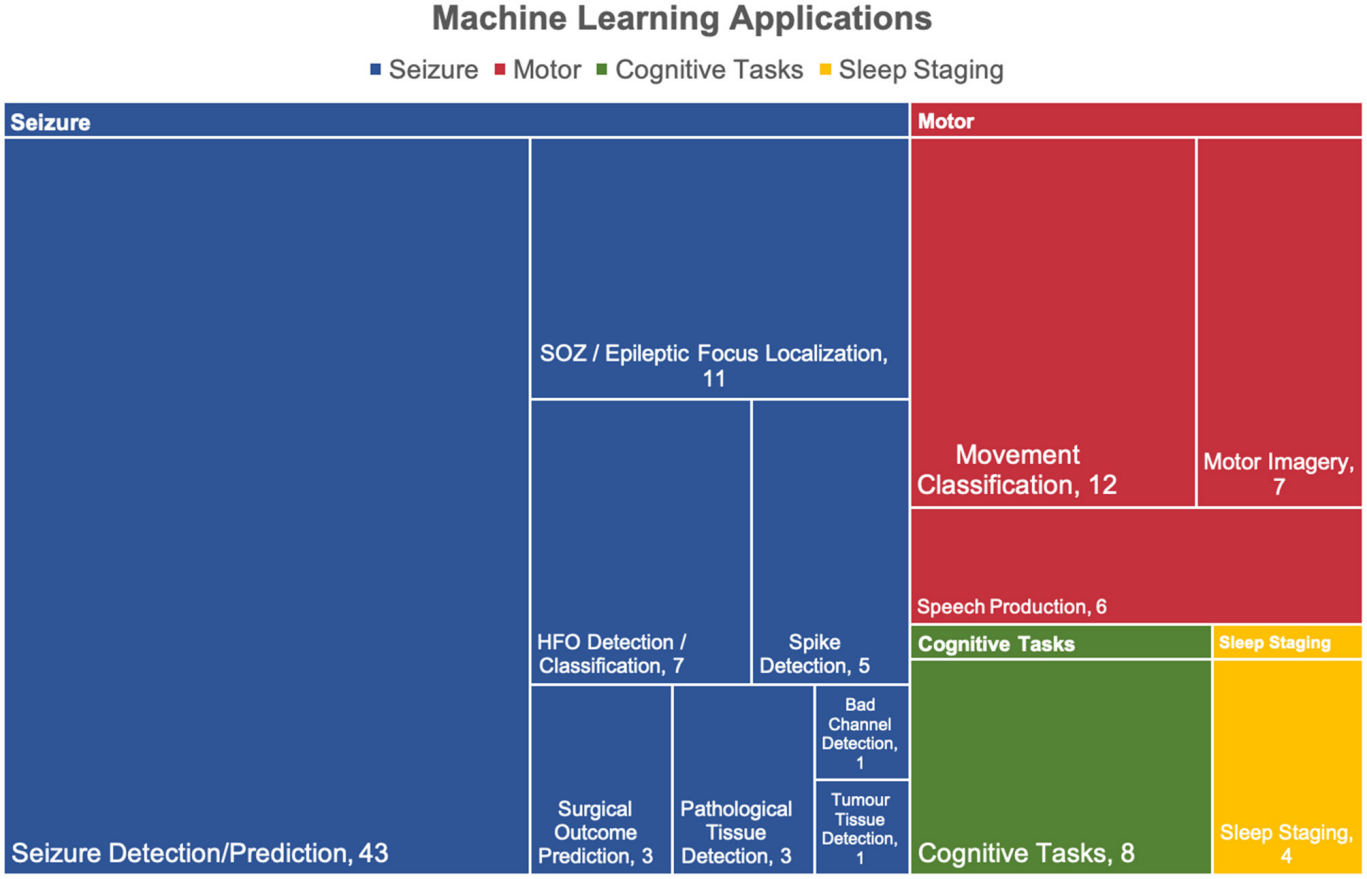
Applications of machine learning in intracranial EEG. The applications were divided into 4 categories: seizure, motor, cognitive tasks. Over half of the articles were relevant to seizures. Four studies had more than one application thereby leading to a total of 111 applicants for 107 articles.

The ML applications for each article were grouped into 4 categories, all illustrated in Figure 5. Four articles employed machine learning for more than one application, thereby leading to 111 counts for 107 articles. The vast majority of applications relate to seizures (74/111, 67%), followed by motor (25/111, 23%), cognitive tasks (8/111, 7%), and sleep staging (4/111, 4%).

#### Applications

The seizure category is divided into 8 subcategories in which seizure detection/prediction (43/74, 58%) and SOZ/epileptic focus localization (11/74, 15%) were the most popular. The motor category is composed of 3 subcategories where movement classification makes up half of the applications (12/25, 48%).

#### Algorithm

The majority of articles reviewed in this study employed standard supervised ML methods only (68/107, 63%), followed by DL only (19/107, 18%). Fourteen articles employed both standard ML and DL in order to compare algorithm performance. Unsupervised learning made up a minority of articles found (6/107, 6%). An illustration of this distribution is presented in Figure 4B. The majority of studies presented results from a single algorithm (78/107, 73%) rather than multiple algorithms (29/107, 27%).

#### Comparing Algorithmic Performance

Performance metrics for each study employing a publicly available dataset are summarized in Table 1. Five public datasets were identified amongst the studies employing ML for seizure detection or prediction. Amongst studies utilizing the same dataset, a variety of different metrics were reported. It is also important to note that while some studies employ all patient data from public datasets, some may only select a subgroup of participant data from a dataset. Therefore, Table 1 provides the specific recording method as reported by the authors.

Amongst the studies utilizing the Freiburg dataset, 11 reported metrics on the entire set of 21 patients (Liu et al., 2012; Zhang et al., 2014, 2015; Yuan et al., 2015; Geng et al., 2016, 2020; Parvez and Paul, 2017; Alickovic et al., 2018; Mahmoodian et al., 2019; Meisel and Bailey, 2019; Yu et al., 2020). Within this group, deep learning methods achieved significantly higher sensitivity for seizure detection compared to standard methods (mean 0.922 vs. 0.980, *p* = 0.019 by Student's *t*-test). Few of these studies, however, reported specificity thereby making a performance comparison based on this metric more difficult. In those using the Freiburg dataset and exclusively investigating standard machine learning (Chua et al., 2011; Liu et al., 2012; Zhang et al., 2014, 2015; Wang and Lyu, 2015; Yuan et al., 2015; Zheng et al., 2015; Geng et al., 2016; Song and Zhang, 2016; Zhang and Parhi, 2016; Parvez and Paul, 2017; Alickovic et al., 2018; Mahmoodian et al., 2019; Yu et al., 2020), SVM was the most popular. Fewer studies employed the Bonn dataset, where deep learning and a vector machine algorithm achieved greater accuracies compared to a standard multilayer perceptron (MLP) with a single hidden layer (Raghu and Sriraam, 2017; Vidyaratne and Iftekharuddin, 2017; Gong et al., 2020). Studies using EPILEPSIAE data all employed standard ML but specificity metrics were not reported for comparison (Manzouri et al., 2018; O'Leary et al., 2018; Ghoroghchian et al., 2020). Those using the Mayo-UPenn show similar accuracies with deep learning and a combination classifier of standard supervised algorithms (Hosseini et al., 2017, 2018; Truong et al., 2018).

Three datasets were employed by studies for SOZ or epileptic focus localization. Two studies used the Bonn and Bern-Barcelona datasets where a deep MLP (more than one hidden layer) (Daoud and Bayoumi, 2020) outperformed a standard SVM (Chen et al., 2017) by achieving greater specificity. The only study that employed the Mayo-UPenn dataset in this context revealed a greater specificity with an LSTM-SVM in comparison to an LSTM alone (Hosseini et al., 2020). This is particularly interesting as it shows that combining a deep learning approach for feature extraction and selection with a standard SVM model may further outperform deep learning alone.

A single publicly available dataset was found amongst the motor studies in the context of motor imagery classification. Three of the 4 studies employing the BCI Competition III dataset split their data into a training and testing group (Demirer et al., 2009; Yang et al., 2012; Rashid et al., 2020) while one only reported their validation accuracy (Li et al., 2015). Amongst the standard ML methods, SVM (Demirer et al., 2009) achieved a greater accuracy than LVQ (Li et al., 2015) and ANN (Yang et al., 2012) in validation sets, but the ANN performed better in testing. This may indicate that although the SVM may performed well in training, it appears to overfit the training data and did not generalize as well as the ANN in this context. However, the most recent study using this dataset employed deep learning and achieved significantly greater accuracies compared to all previous standard ML methods in both training and testing (Rashid et al., 2020).

All studies involved in cognitive tasks or sleep staging used unique datasets and therefore cannot be effectively compared based on classification performance. Amongst the cognitive task studies, two compared different types of algorithm within their own dataset. Though detailed statistical comparison is not possible, deep learning appeared to outperform linear regressions and SVM (Arora et al., 2018). A second study revealed that a combination of LSTM and CNN outperformed CNN alone thereby further probing the potential of combined approach (RaviPrakash et al., 2020). No sleep staging studies were reported to compare different algorithms.

### Influence of Feature Selection

In general, studies focusing on seizure detection and prediction applied time-frequency representations of raw EEG signals. This was accomplished with bandpass filtering or signal decomposition using Fourier or wavelet transforms. Less commonly other signal decomposition methods were also used for this purpose, such as the Stockwell transform (Geng et al., 2020), harmonic wavelets (Vidyaratne and Iftekharuddin, 2017) or empirical mode decomposition (EMD) (Zheng et al., 2015). These transforms yield frequency-domain representations of the signal, namely the amplitudes of various frequency ranges in a given signal segment or epoch. These amplitudes were most often used to calculate statistical features such as the mean, variance, and standard deviation (Alickovic et al., 2018; Meisel and Bailey, 2019), as well as higher-order statistical features including skew and kurtosis (Mirowski et al., 2009; Hosseini et al., 2017). Amplitude at various frequencies were also be used to calculate the envelope (Wang and Lyu, 2015; Meisel and Bailey, 2019) (a function enclosing the peaks of the signal, or instantaneous amplitude), or line length (Baud et al., 2018) to capture spikes. Several algorithms in our dataset used bivariate or multivariate (i.e., features calculated from pairs or groups of electrodes) features including cross-correlation and phase-locking value between pairs of signals, as well as higher-order measures of synchrony such as non-linear interdependence, dynamical entrainment, and entropy in difference of phases (Mirowski et al., 2009; O'Leary et al., 2018).

Signal entropy is a measure of the predictability of a signal–that is, disordered and unpredictable signals have high entropy and vice versa. Studies reviewed herein used entropy of various time series, in particular the raw EEG signal, frequency and power spectra, phase, as well as multi-electrode signals (Hosseini et al., 2017; Vidyaratne and Iftekharuddin, 2017; Mahmoodian et al., 2019). A closely related measure to entropy is fractal dimension, in that the latter measures the “roughness” of a signal at different scales. The fractal dimension of frequency- or time-domain signals can be estimated using box-counting methods, as well as the more formal Hausdorff dimension.

SOZ localization depends on the detection and analysis of signal phenomena such as HFOs, phase-amplitude coupling (PAC), and interictal epileptiform discharges (IED). In particular, HFOs have been proposed as epileptogenic biomarkers with emerging prognostic utility in cortical resection outcomes (Jacobs et al., 2010; Frauscher et al., 2017). These events are first detected in raw EEG signals using well-validated detectors in the literature, such as the line-length and Cimbalnik-Stead HFO detectors. The rate of these events in specific electrodes are used to localize the epileptic focus, either as the sole feature (Sumsky and Santaniello, 2019) or in an ensemble with other first-order or abstract features (Klimes et al., 2019).

Features for speech production, motor imagery, and cognitive tasks differed in key respects with those for seizure prediction. While high-frequency oscillations in high-gamma (70-135Hz) and ripple (135-200Hz) bands were most useful in seizure prediction (Pan et al., 2018), features sources from lower frequencies, such as theta (Livezey et al., 2019), alpha (Andrade et al., 2017; Hosseini et al., 2020), and beta bands (Yang et al., 2012) were most useful for speech, motor and cognitive tasks. In addition, features for these three categories generally focused on amplitude, spectral power and its transformations rather than measures of disorder like entropy and fractal dimension (Pan et al., 2018; Xie et al., 2018; Pailla et al., 2019; Thomas et al., 2019). In particular, the normalized (or Z-transformed) log-spectral power obtained from a morlet wavelet transform is commonly used in speech and cognitive tasks (Ikeda et al., 2014; Schrouff et al., 2016; Kragel et al., 2017; Arora et al., 2018; Weidemann et al., 2019).

Hand-selected feature sets through these various methods may be ensemble-optimized to reduce dimensionality and redundancy, thereby making downstream classification more accurate and computationally efficient. Parallel arrays of time-varying features may be subject to dimensionality reduction using principal component analysis (PCA) or its variants as a data pre-processing step (Tomlinson et al., 2017; O'Leary et al., 2018; Yu et al., 2020). Some algorithms rely on feature selection using some optimization algorithm or neural network (Firpi et al., 2007b; Antoniades et al., 2017; Abou Jaoude et al., 2020), or even generating artificial features *de novo* using a genetic algorithm (Firpi et al., 2007a; Yang et al., 2012; Sathish et al., 2017).

Measures such as fractal dimension and higher-order moments have unintuitive physical correlates and may not correlate with underlying biological signals. Thus, we compared “higher-order” feature selection algorithms with approaches with physically interpretable features to determine if differences in model performance arise. Studies that used higher-order feature-selection algorithms were defined as those in which the feature selection/pre-processing workflows contained one or more of: i) non-standard time-frequency transforms; ii) dimensionality reduction of feature space; iii) abstract feature selection algorithms; or iv) abstract features (entropy, fractal dimension, etc.). When comparing seizure prediction/detection algorithms that were tested on all 21 patients from the Freiburg iEEG dataset, “standard” and “higher-order” feature selection approaches did not differ in overall sensitivity (mean 0.966 vs. 0.930, *p* = 0.1696 by Student's *t*-test). This suggests that while higher-order input features have few biological and physical correlates, this penalty to interpretability does not necessarily translate into better model performance.

## Discussion

The study of iEEG provides unprecedented insight into the neural code, revealing the underpinnings of a variety of physiological and pathological brain processes. At present, the gold standard of iEEG analysis involves extraction of features through digital signal processing and analyses by human experts. However, ML is rapidly becoming a viable tool that may facilitate better understanding of intracranial data. In the current review, we describe the landscape of ML applied to iEEG. We also compare and contrast various methods and provide a synopsis of relative advantages and pitfalls.

### Seizure

Machine learning applications to iEEG predominantly relate to epilepsy. The majority of studies reviewed herein focused on classification of signals as seizures or non-seizure events, while others employed ML for seizure onset prediction. In the latter, the algorithm identifies patterns in the pre-ictal signal in order to pre-emptively predict seizure onset. This is a particularly challenging task for humans to accomplish and therefore an ideal opportunity for the implementation of AI tools (Assi et al., 2019).

Deep learning algorithms demonstrate the best performance in the classification of seizures and SOZ localization. Unlike an ML-informed approach, current practices of seizure detection are susceptible to inter-rater variability. A recent study studying seizure detection from ECoG signal revealed an interrater agreement of only 79% (Quigg et al., 2015). This supports the potential role of using ML-powered algorithms to better inform experts in complex cases thereby reducing variability.

Another benefit of ML-based tools is the ability to integrate multiple data streams including EEG, electromyography (EMG), accelerometry (ACM), electrocardiography (ECG), near-infrared spectroscopy (NIRS), video, and electrodermal activity (EDA) inputs beyond what can be achieved by a human interpreter (Ulate-Campos et al., 2016). This type of approach has also been applied for other applications relevant to neurology such as early detection of Parkinson's disease (Prashanth et al., 2016), falls (Nahiduzzaman et al., 2020) and identification of cortical dysplasia (Mo et al., 2019). One of the studies reviewed utilized this multidimensional approach and achieved an accuracy of 95% for surgical outcome prediction in epilepsy patients, albeit on an in-house dataset (Memarian et al., 2015).

Enhanced SOZ localization is an emerging area for increased ML utilization. Indeed, in medically-refractory epilepsy, post-operative seizure freedom rates remain in the range of 50–70% despite multimodal analytic tools to guide localization and resection of the putative epileptogenic zone (Englot et al., 2011, 2012). Accurate localization of the SOZ also remains the strongest predictor of positive surgical outcome (Jacobs et al., 2010). Through network-based analytic approaches, ML algorithms can provide more detailed localizations of seizure onset zones based on higher-order patterns not immediately apparent to experts, with the potential to improve post-operative outcomes. Studies reviewed herein demonstrate accuracies in excess of 90% using deep learning and 80% with standard ML (Chen et al., 2017; Daoud and Bayoumi, 2020; Hosseini et al., 2020). A growing body of literature also supports the use of ML in identifying spectral patterns such as high-frequency oscillations that may further refine SOZ localization (Jacobs et al., 2010; Weiss et al., 2019; Si, 2020).

Beyond epilepsy, the breadth of ML use in neurosurgical outcome prediction spans multiple fields from neoplasms to spinal disease to arteriovenous malformations, with promising success. (Senders et al., 2018) Such applications of ML may not only guide the decision-making process of neurosurgical teams when selecting surgical candidates, but may also reveal patterns previously unknown to influence surgical outcome.

Despite the significant potential of deep learning, there are several challenges to its clinical deployment. First, deep learning generally requires a significantly larger pool of data compared with standard ML algorithms, and datasets of this size may be clinically unavailable. A second limitation of deep learning relates to its status as a “blackbox” algorithm whereby the underlying decision-making process of the algorithm cannot be readily understood. A great deal of contemporary literature has examined this subject and is producing algorithmic approaches to explain such “blackbox” models. Examples include game theoretic “Shapley” values and permutation importance which provide insight through backpropagation of altered to retrospectively determined feature importance (Wong et al., 2019; Sundararajan and Najmi, 2020). Conversely, standard ML remains more transparent without the use of these complex middle-ground interpreters. Given the reported performance advantages of deep learning, we suggest that an area for substantial future research should relate to the development of clinical tools that may improve inherent sample sizes (such as the development of large, centralized databases). We also recommend that future work should also focus on deeper development of backpropagation tools to explain model predictions.

Interestingly, the realm of neuro-oncology may also be impacted by these advanced AI technologies. A study reviewed herein presented a novel proof of concept using ML to differentiate healthy tissue from brain tumor tissue during awake craniotomy (Boussen et al., 2016). Although the gold standard of intraoperative mapping remains direct cortical stimulation, Boussen et al. were able to discriminate tumoral tissue from eloquent brain using an ANN based on spectral data from ECoG electrodes with a classification accuracy of 93.6%. The finding of profound spectral alteration within tumoral tissue when compared to healthy brain may serve as a useful intraoperative adjunct in awake surgery.

### Motor

A significant number of machine learning applications to iEEG relate to motor tasks. Our analysis of the model performances in this context provided a reminder on the importance of model generalizability. Although Demirer et al.'s SVM (Demirer et al., 2009) showed better validation metrics for motor imagery classification with the BCI Competition III dataset compared to Yang et al.'s ANN (Yang et al., 2012), the final testing accuracy was significantly lower. This finding poses a critical reminder with regards to overfitting. When a validation set is used to explicitly optimize a model, the metrics must be interpreted with caution as they may provide an overly optimistic estimate of model performance, as seen in the Demirer study. Therefore, whenever possible, performance should be evaluated on an unseen testing set once the model has been finalized to accurately judge generalization performance. As it relates to the classification of motors tasks, given the small number of studies for comparison, it is not possible to conclude whether an ANN or SVM would be superior in terms of motor classification tasks. However, it does underscore the importance of using unseen data to generate an unbiased estimate of model performance.

The majority of movement classification studies employ algorithms to differentiate motor tasks such as upper limb movement (Yanagisawa et al., 2009; McMullen et al., 2014; Thomas et al., 2019) and hand or finger gesturing (Scherer et al., 2009; Pan et al., 2018; Xie et al., 2018; Pailla et al., 2019; Zhu et al., 2020) based on electrophysiological recordings. One study used a similar paradigm but trained their algorithm to differentiate rest, movement intention and movement execution (Combrisson et al., 2017). By distinguishing the vital steps of motor preparation based on neural correlates, ML in these cases can provide insight on the importance of specific features such as oscillatory phase and amplitude in understanding motor function. On the other hand, motor imagery tasks require participants to imagine performing a specific action. In these studies (Hill et al., 2006; Shenoy et al., 2008; Demirer et al., 2009; Yang et al., 2012; Li et al., 2015; Andrade et al., 2017; Rashid et al., 2020), algorithms were trained to identify when a participant imagined a specific action. Within the examined studies, we see that modern ML approaches can achieve a significant degree of accuracy–up to 98%-in these classification tasks.

The relevance of combining iEEG signals with ML in these domains can relate to better tailoring of neuroprostheses to individual patient dynamics. Whereas, current myoelectric prostheses follow a “one-size-fits-all” approach, the adaptability of ML-based neuroprotheses could learn from the patient's individual movements and adapt to become more responsive and precise over time. Indeed, a recent survey of upper limb prothesis users reveals a desire for adaptable grip strength and individual finger and thumb movements (Cordella et al., 2016). Just as humans learn to adapt these composites of motor movements, a brain-computer interface (BCI) powered by machine learning may be able to learn to adapt as the user interacts with the environment. Furthermore, recent studies indicate that BCI prosthetics may play a role in reducing phantom limb pain (Yanagisawa et al., 2020). The underlying mechanism for this change has yet to be elucidated although it is believed to be related to neuroplasticity in the sensoricortex.

The last sub-category of motor tasks relates to speech production (Ramsey et al., 2018; Livezey et al., 2019). Here, participants were asked to articulate sounds, phenomes or words presented on a screen. Algorithms were trained to differentiate these sounds based on iEEG. Unlike the traditional myoelectric or keyboard-based voice prothesis, neuroprostheses involve a BCI that can extract EEG signals to reconstruct the patient's speech. In this review, a single study (Makin et al., 2020) employed ML to reconstruct sentences from iEEG signal in a small number of participants, demonstrating a word error rate (measure of incorrect predictions) of 3% compared to the professional level of 5% (Xiong et al., 2017). Though these applications are clearly within their infancy, speech prostheses powered by ML may similarly learn from each individual patient's speech patterns to improve speech reconstruction performance with use.

### Cognitive Tasks

The study of cognitive performance is unique in that the neural circuitry and activity patterns remain poorly understood. This has limited the development of neuromodulatory interventions, such as deep brain or closed-loop stimulation that could potentially treat deficits in subdomains of cognition. A key role for ML therefore lies in its potential to identify activity distributed across neural assemblies related to specific tasks.

Within the studies we report, memory was the most common cognitive task studied (Kragel et al., 2017; Arora et al., 2018; Derner et al., 2018; Saboo et al., 2019; Weidemann et al., 2019). Three studies employed standard ML including SVM and LR although no comparisons were made between different types of algorithm. Derner et al. (2018) obtained average classification accuracies for memory recall in the range of 60%; similarly, Kragel et al. (2017) presented AUCs of 0.59 and 0.68 with a classifier able to estimate memory task success during retrieval and encoding, respectively. Weidemann et al. (2019) developed LR models that performed better than chance while also noticing that their memory performance prediction classifiers could generalize to unrelated lists. This supports that their classifier is trained to distinguish episodic memory without discriminating for sematic content (Weidemann et al., 2019). This has significant implications for the future development of closed-loop stimulation powered by ML which may be able to predict memory performance across a breadth of contexts. Arora et al. (2018) is the only study that employed deep learning for memory task performance classification and showed that LSTM (mean AUC 0.72) modestly outperformed SVM (mean AUC 0.68) which itself significantly outperformed LR (mean AUC 0.59). Hence, there remains debate whether a modest improvement in performance outweighs the lack of transparency of LSTM in comparison with SVM.

The complexity of neural activity during cognitive tasks is reflected in the relatively modest improvements in algorithm classification performance when compared to chance. However, despite these challenges, these models have shown practical utility in closed-loop stimulation for memory (Ezzyat et al., 2018).

Other studies in the cognitive realm employed machine learning for unique purposes. Saboo et al. (2019) employed a novel approach by comparing an unsupervised clustering model with LDA and SVM to identify active electrodes during a verbal memory task. Their unsupervised method significantly outperformed standard supervised ML reaching a sensitivity of 97% and specificity of 92.9% (Saboo et al., 2019). However, as the ground truth for these labels was defined by expert reviewers, the authors were unable to compare their performance with human experts as the algorithm aimed to recreate the human decisions. Nonetheless, this method provides a much faster alternative to the highly time-consuming and subjective process of human expert labeling (Saboo et al., 2019).

In another, SVMs were used to differentiate whether participants were performing a mathematical or a memory task and achieved best accuracies of 89.51, 87.18 and 76.80% for 3 participants (Schrouff et al., 2016). Another study by Hermiz et al. (2018) employed numerous approaches with a logistic regression to differentiate different types of auditory and visual stimuli achieving accuracies in the 80 to 95% range. Lastly, RaviPrakash et al. (2020) developed a deep learning language comprehension algorithm able to differentiate whether participants are actively listening to a story, or listening to broadband noise. The authors achieved a greater performance with a combination algorithm of LSTM and CNN as opposed to CNN alone, with a classification accuracy of 83%. Although several studies in the field of cognition revealed a better performance of DL algorithms, the small sample size limits definitive conclusions.

### Sleep Staging

Studies identified in this review employed supervised (Kremen et al., 2017; Rutigliano et al., 2018; Principe et al., 2019) or unsupervised (Kremen et al., 2019) machine learning to differentiate sleep stages in patients diagnosed with epilepsy. Kremen et al. (2017) employed SVM to classify awake states and slow wave sleep with an accuracy of 97.8% and 89.4% in healthy and epileptic tissue, respectively. A more recent study by this group (Kremen et al., 2019) obtained a 94% accuracy with unsupervised learning for more complex classification of three groups: awake states, N2 and N3 sleep. Rutigliano et al. (2018) employed an ANN with a single hidden layer to classify wakefulness and non-REM sleep with an accuracy of 98.57%. As the labels were defined by a human neurologist, a comparison to expert performance cannot be established.

The primary advantage of ML in this context is the speed of classification and accuracy of sleep-stage detection which would be of importance to a variety of neuromodulatory therapeutics that function during specific stages of sleep (Kremen et al., 2019). An example of such an approach includes closed-loop auditory stimulation during sleep for epilepsy (Fattinger et al., 2019). Furthermore, such tools can lessen the healthcare burden related to long waiting periods for patients awaiting formal sleep studies (Rotenberg et al., 2010).

### Limitations of ML Algorithms

The literature search conducted herein revealed that multiple different types of ML algorithms can be employed in the context of iEEG analysis and classification for variety of applications. However, each algorithm faces its own set of limitations. Generally, standard algorithms including SVM, KNN, and decisions trees may not be suitable for very large datasets. Training an SVM also involves numerous parameters which can be a lengthy process to optimize, and they can be also be biased if the number of features provided is greater than the number of samples in the training set. KNNs also perform poorly when using a large number of features and they are particularly sensitive to outliers or missing values. Decision trees can become hypersensitive to its input data whereby small changes in the input can have drastic effects on the output classification. As an ensemble of decision trees, RFs are can be limited by their interpretability and are often considered a black box. Similarly, deep learning models are generally criticized for their lack of interpretability, although novel methods attempting to elucidate how these complex multilayered models make decisions are discussed in the literature (Montavon et al., 2018). A thorough understanding of the dataset, as well as the goals of the ML algorithm are critical to select appropriate models that are best suited to data.

### Future Recommendations

Exploring the current ML literature, we identified three recommendations which may be useful for future work in ML and iEEG. First, we believe that future studies should aim to not only report performance metrics but provide a specific reference point for comparison–whether the best previous ML performance or contemporary expert performance. Such comparisons can help ground clinical interpretation of the data and identify the potential practical utilizations of ML in routine clinical practice. Second, large datasets representative of the target population may be advantageous over small datasets as these are more likely to generalize well when applied to new patients. This reinforces the importance of internal and external validation to ensure that algorithms can perform equally well with previously unseen data. Third, it may be advantageous to employ explainable ML algorithms rather than “black box” models in order to shed light on algorithms' decision-making processes. More transparent models may not only offer new knowledge on important features of disease but may also improve the trust from the medical community to employ these models clinically. A study centered around classification performance may employ a more convoluted algorithm while sacrificing interpretability while one aiming to provide new knowledge on the classification process may sacrifice some performance for interpretability.

## Conclusions

Artificial intelligence is expected to have a significant impact on the future practice of neurosurgery. By identifying complex and hidden patterns that may be inconspicuous to human counterparts, ML algorithms may not only offer more accurate and more rapid tools to assist decision making but may also elucidate the underlying mechanisms involved in a number of neurological disorders.

This study achieved both of our aims: to assess the current landscape of ML in iEEG, and to examine the relative benefits and limitations of ML approached as they related to neurosurgical problems. We conducted a systematic search across three databases encompassing the fields of medicine, science and engineering and identified 107 articles. We nonetheless recognize that some articles may have been missed within our systematic search considering the rapid rate at which papers within the realm of AI are currently being published.

The studies reviewed herein reveal a promising future of AI in iEEG by shedding light on the variety of ML algorithms that can be employed to classify data in the context of seizures, motor tasks, cognitive tasks and sleep. Of these, supervised algorithms were most commonly employed with most articles published in the realm of seizures and epilepsy. The merits of using ML to tackle neurosurgical problems are broad including the development of more personalized neuroprostheses, standardizing seizure detection compared to human analyses, predicting surgical outcomes, and improving the speed and classification of sleep stages.

As AI becomes increasingly utilized in medicine and surgery, we intend for this work to serve as a resource to aid in the interoperation and development of relevant technologies employing ML in the context of iEEG analysis.

## Data Availability Statement

The original contributions presented in the study are included in the article/Supplementary Material, further inquiries can be directed to the corresponding author/s.

## Author Contributions

NM and NW led the study and conducted the literature search, review and analyses. FZ conducted the statistical analyses and contributed to extracting content from the literature. SW and LE provided extensive input on machine learning. SW, HS, KM, LE, and GI assisted and provided input throughout the review process. NM, NW, and FZ wrote the manuscript with input and feedback from all authors. GI was in charge of the overall direction and planning. All authors contributed to the article and approved the submitted version.

## Conflict of Interest

The authors declare that the research was conducted in the absence of any commercial or financial relationships that could be construed as a potential conflict of interest.

## Publisher's Note

All claims expressed in this article are solely those of the authors and do not necessarily represent those of their affiliated organizations, or those of the publisher, the editors and the reviewers. Any product that may be evaluated in this article, or claim that may be made by its manufacturer, is not guaranteed or endorsed by the publisher.
