# Supplementary material for: Decoding Intracranial EEG With Machine Learning: A Systematic Review
Source: Front Hum Neurosci. 2022 Jun 27;16:913777. doi: 10.3389/fnhum.2022.913777 (PMC9271576; doi:10.3389/fnhum.2022.913777)
Supplement: Supplementary file 1 [file Data_Sheet_1.docx]

**Supplemental Material 1: Search strategy for systematic review of artificial intelligence in intracranial EEG.**

| **Database: Web of Science (all document types, all languages, 1900-2020)** | | |
| --- | --- | --- |
| Search # | Search Strategy | # of articles |
| 1 | TI=("machine learning")  OR  AB=("machine learning")  OR  AK=("machine learning") | **131,672** |
| 2 | TI=("deep learning")  OR  AB=("deep learning")  OR  AK=("deep learning") | **51,806** |
| 3 | TI=("artificial intelligence")  OR  AB=("artificial intelligence")  OR  AK=("artificial intelligence") | **52,635** |
| 4 | TI=("neural network$")  OR  AB=("neural network$")  OR  AK=("neural network$") | **333,143** |
| 5 | #4 OR #3 OR #2 OR #1 | **496,586** |
| 6 | TI=("stereoelectroencephalography" OR "stereotactic electroencephalography" OR "stereotactic EEG" OR sEEG)  OR  AB=("stereoelectroencephalography" OR "stereotactic electroencephalography" OR "stereotactic EEG" OR sEEG)  OR  AK=("stereoelectroencephalography" OR "stereotactic electroencephalography" OR "stereotactic EEG" OR sEEG) | **1,055** |
| 7 | TI=(electrocorticography OR  ECoG)  OR  AB=(electrocorticography OR ECoG)  OR  AK=(electrocorticography OR ECoG) | **8,778** |
| 8 | TI=("intracranial EEG" OR iEEG OR "intracranial electroencephalography")  OR  AB=("intracranial EEG" OR iEEG OR "intracranial electroencephalography")  OR  AK=("intracranial EEG" OR iEEG OR "intracranial electroencephalography") | **2,015** |
| 9 | #8 OR #7 OR #6 | **11,566** |
| 10 | #9 AND #5 | **290** |

| **Database: OVID MEDLINER (1860-current, all languages)** | | |
| --- | --- | --- |
| Search # | Search Strategy | # of articles |
| 1 | Exp machine learning/ | **19,324** |
| 2 | Exp deep learning/ | **2,576** |
| 3 | Exp artificial intelligence/ | **98,444** |
| 4 | Exp neural networks, computer/ | **27,773** |
| 5 | 1 or 2 or 3 or 4 | **98,444** |
| 6 | (stereoelectroencephalography or "stereotactic electroencephalography" or "stereotactic EEG" or sEEG).mp. | **964** |
| 7 | Exp electrocorticography/ | **986** |
| 8 | ECoG.mp. | **7,815** |
| 9 | ("intracranial EEG" or iEEG or “intracranial electroencephalography”).mp. | **1,636** |
| 10 | 6 or 7 or 8 or 9 | **10,608** |
| 11 | 5 and 10 | **159** |

| **Database: IEEE Xplore (1872-2021)** | | |
| --- | --- | --- |
| Search # | Search Strategy | # of articles |
| 1 | (((("All Metadata":"machine learning") OR "All Metadata":"deep learning") OR "All Metadata":"artificial intelligence") OR "All Metadata":"neural network*") | **389,817** |
| 2 | ((((((((("All Metadata":stereoelectroencephalography) OR "All Metadata":"stereotactic electroencephalography") OR "All Metadata":"stereotactic EEG") OR "All Metadata":sEEG) OR "All Metadata":"electrocorticography") OR "All Metadata":ECoG) OR "All Metadata":"intracranial EEG") OR "All Metadata":iEEG) OR “All Metadata”:””intracranial electroencephalography”) | **1,109** |
| 3 | 1 AND 2 | **225** |
