# Supplementary material for: Decoding Intracranial EEG With Machine Learning: A Systematic Review
Source: Front Hum Neurosci. 2022 Jun 27;16:913777. doi: 10.3389/fnhum.2022.913777 (PMC9271576; doi:10.3389/fnhum.2022.913777)
Supplement: Supplementary file 2 [file Data_Sheet_2.docx]

**Supplementary Material 2: Data extracted from literature on artificial intelligence in intracranial EEG.**

| **Application** | | | **Dataset** | **Authors** | **Recording Method** | **Machine Learning** | | | **Features** | **Journal Category** |
| --- | --- | --- | --- | --- | --- | --- | --- | --- | --- | --- |
|  |  |  |  |  |  | Category | Models | Best Al Performance |  |  |
| Seizure | Seizure Detection / Prediction | | Freiburg | Yu et al., 2020 | Not specified | Standard | LDA | 87.7% (ss) | Higher order | Engineering |
|  |  |  |  | Geng et al., 2020 | Not specified | Deep | LSTM | 98.09% (ss), 98.69% (sp) | Higher order | Engineering |
|  |  |  |  | Lian et al., 2020 | Not specified | Standard & Deep | JGRN, CNN, GCNN, SVM, XGB | 95.67% (acc), 83.68% (rec), 92.52% (prec), 85.56% (F1) | Higher order | Engineering |
|  |  |  |  | Truong et al., 2019 | Not specified | Standard & Deep | GAN-NN, CNN | 88.86 (auc) | Higher order | Engineering |
|  |  |  |  | Meisel et al., 2019 | Strips/grids | Standard & Deep | KNN, SVM, DT, DNN | Not reported | Higher order | Medical |
|  |  |  |  | Mahmoodian et al., 2019 | Not specified | Standard | SVM | 96.8% (acc), 95.8% (ss), 96.7% (sp) | Higher order | Medical |
|  |  |  |  | Alickovic et al., 2018 | Not specified | Standard | RF, SVM, MLP, KNN | 100% (acc) | Higher order | Engineering |
|  |  |  |  | Parvez et al., 2017 | Not specified | Standard | SVM | 95.4% (acc) | Standard | Engineering |
|  |  |  |  | Zhang et al., 2016 | Not specified | Standard | SVM | 100% (ss), 0.032 (FPR) | Standard | Engineering |
|  |  |  |  | Geng et al., 2016 | Not specified | Standard | WNN | 96.72% (ss), 98.91 (sp) | Standard | Engineering |
|  |  |  |  | Song et al., 2016 | Not specified | Standard | ELM, SVM | 85.73% (acc) | Higher order | Medical |
|  |  |  |  | Zheng et al., 2015 | Not specified | Standard | SVM | 92% (ss) | Higher order | Medical |
|  |  |  |  | Wang et al., 2015 | Not specified | Standard | SVM | 98.8% (ss) | Higher order | Engineering |
|  |  |  |  | Yuan et al., 2015 | Not specified | Standard | KCRC | 94.41% (ss), 96.97% (sp),  96.87% (acc) | Higher order | Engineering |
|  |  |  |  | Zhang et al., 2015 | Not specified | Standard | RVM | 92.94% (ss), 97.47% (sp),  97.57% (acc) | Higher order | Engineering |
|  |  |  |  | Zhang et al., 2014 | Not specified | Standard | LDA | 89.33% (ss), | Higher order | Medical |
|  |  |  |  | Liu et al., 2012 | Not specified | Standard | SVM | 94.46% (ss), 95.26% (sp), 95.33 (acc) | Standard | Engineering |
|  |  |  |  | Chua et al., 2011 | Not specified | Standard | QDA | 78% (ss) | Standard | Medical |
|  |  |  |  | Liu et al., 2009 | Strips/grids & Depth electrodes | Deep | PFNN | 93.75% (ss), | Standard | Engineering |
|  |  |  |  | Mirowski et al., 2009 | Strips/grids & Depth electrodes | Standard & Deep | LR, CNN, SVM | 71% (ss) | Higher order | Medical |
|  |  |  | Bonn | Gong et al., 2020 | Not specified | Standard & Deep | PNN, LVQ, ENN | 99.79% (acc CE), 98.96% (acc DE), 83.13% (acc CD), 98.75% (acc CD-E), 85.75% (acc CDE) | Standard | Engineering |
|  |  |  |  | Vidyaratne et al., 2017 | Not specified | Standard | RVM | 99.8% (acc CD-E), 99% (ss CD-E), 100% (sp CD-E) | Higher order | Engineering |
|  |  |  |  | Raghu et al., 2017 | Strips/grids & Depth electrodes | Standard | MLP | 97.68% (acc CE), 94.56% (acc DE), 84.58% (acc CDE), 57.8% (acc CD) | Higher order | Engineering |
|  |  |  | EPILEPSIAE | Ghoroghchian et al., 2020 | Not specified | Standard | RF | 0.89 (auc) | Standard | Engineering |
|  |  |  |  | Manzouri et al., 2018 | Strips/grids & Depth electrodes | Standard | SVM, RF | 0.98 (auc) | Higher order | Medical |
|  |  |  |  | O’leary et al., 2018 | Not specified | Standard | SVM | 97.7% (ss), 0.185 (fdr) | Higher order | Engineering |
|  |  |  | Mayo-UPenn | Hosseini et al., 2018 | Strips/grids & Depth electrodes | Standard | SVM, KNN, MLP, Combination classifier | 97% (acc), 98% (ss), 96% (sp), 4% (fpr), 2% (fnr) | Higher order | Medical |
|  |  |  |  | Hosseini et al., 2017 | Strips/grids | Standard & Deep | CNN, stacked autoencoder, RF, SVM, MLP | 96% (acc), 97% (ss), 97% (prec), 0.05 (fpr), 0.03 (fnr) | Higher order | Engineering |
|  |  |  | Bern-Barcelona | Sathish et al., 2017 | Not specified | Standard | KNN, SVM, FFNN | 99.6% (acc) | Higher order | Multidisciplinary |
|  |  |  | Freiburg  &  Mayo-Upenn | Truong et al., 2018 | Not specified | Deep | CNN, BW-CNN, XNOR-Net, Integer-Net | 94.7% (acc; Freiburg), 96.18% (acc; M-UP cross-validation), 88.81% (acc; M-UP testing) | Standard | Engineering |
|  |  |  | Own data | Burrello et al., 2020 | Strips/grids & Depth electrodes | Standard & Deep | RF, SVM, MLP, LSTM, HD | n/a | Higher order | Engineering |
|  |  |  |  | Kiral-Kornek et al., 2018 | Not specified | Deep |  |  | Standard | Medical |
|  |  |  |  | Shoaran et al., 2018 | Not specified | Standard | XGB, KNN, SVM, LR |  | Standard | Engineering |
|  |  |  |  | Baud et al., 2018 | Strips/grids & Depth electrodes | Unsupervised | NNMF |  | Higher order | Medical |
|  |  |  |  | Khambhati et al., 2017 | Strips/grids | Unsupervised | NNMF |  | Higher order | Medical |
|  |  |  |  | Kharbouch et al., 2011 | Not specified | Standard | SVM |  | Standard | Medical |
|  |  |  |  | Ayala et al., 2011 | Strips/grids | Standard | ANN |  | Standard | Medical |
|  |  |  |  | Chan et al., 2008 | Strips/grids & Depth electrodes | Standard | SVM |  | Standard | Medical |
|  |  |  |  | Firpi et al., 2006 | Strips/grids | Standard | KNN |  | Higher order | Engineering |
|  |  |  |  | Petrosian et al., 1999 | Depth electrodes | Deep | RNN |  | Standard | Engineering |
|  | SOZ / Epileptic Focus Localization | | Bonn  &  Bern-Barcelona | Daoud et al., 2020 | Not specified | Deep | MLP | Bern-Barcelona  93.21% (acc), 90.50% (ss), 95.92% (sp), 95.68% (PPV), 90.99% (NPV)  Bonn  96% (acc), 93% (ss), 99% (sp), 98.9% (PPV), 93.4% (NPV) | Standard | Engineering |
|  |  |  |  | Chen et al., 2017 | Not specified | Standard | SVM | Bern-Barcelona  83.07% (acc), 83.05% (ss), 83.09% (sp), 83.09% (PPV), 83.05% (NPV)  Bonn  88.00% (acc), 92.24% (ss), 83.76% (sp), 85.03% (PPV), 91.52% (NPV) | Higher order | Engineering |
|  |  |  | Mayo-Upenn | Hosseini et al., 2020 | Depth electrodes | Standard & Deep | LSTM, LSTM+SVM | 98% (acc), 96 (ss), 97% (sp) | Standard | Medical |
|  |  |  | Own data | Akter et al., 2020 | Not specified | Standard | SVM | n/a | Higher order | Multidisciplinary |
|  |  |  |  | Karthick et al., 2020 | Depth electrodes | Standard & Deep | KNN, LR, MLP, SVM, RF, Rotation Forest |  | Higher order | Medical |
|  |  |  |  | Klimes et al., 2019 | sEEG | Standard | SVM |  | Higher order | Medical |
|  |  |  |  | Sumsky et al., 2020 | Not specified | Standard | SVM, LR |  | Standard | Engineering |
|  |  |  |  | Varatharajah et al., 2018 | Depth electrodes | Standard | SVM |  | Higher order | Engineering |
|  |  |  |  | Grinenko et al., 2018 | sEEG | Standard | SVM |  | Standard | Medical |
|  |  |  |  | Elahian et al., 2017 | Strips/grids | Standard | LR |  | Standard | Medical |
|  | HFO Detection / Classification | | Own data | Sciaraffa et al., 2020 | Not specified | Standard | LDA, LR, SVM, KNN, RF | n/a | Standard | Medical |
|  |  |  |  | Zhao et al., 2020 | sEEG | Deep | CNN (Restnet101) |  | Standard | Medical |
|  |  |  |  | Lai et al., 2019 | Strips/grids | Deep | CNN |  | Standard | Engineering |
|  |  |  |  | Wu et al., 2018 | Strips/grids | Unsupervised | FCM-QEM- Based EM-GMM |  | Higher order | Engineering |
|  |  |  |  | Jrad et al., 2017 | Not specified | Standard | SVM |  | Higher order | Engineering |
|  |  |  |  | Firpi et al., 2007 | Strips/grids, Depth electrodes | Standard | KNN |  | Higher order | Engineering |
|  | Spike Detection | | Own data | Abou Jaoude et al., 2020 | Strips/grids | Deep | CNN | n/a | Standard | Medical |
|  |  |  |  | Makaram et al., 2020 | Depth electrodes | Standard | SVM |  | Higher order | Medical |
|  |  |  |  | Medvedev et al., 2019 | Not specified | Deep | LSTM |  | Standard | Multidisciplinary |
|  |  |  |  | Antoniades et al., 2017 | Not specified | Deep | CNN |  | Standard | Engineering |
|  |  |  |  | Hellmann et al., 1999 | Strips/grids | Standard | Perceptron, ANN |  | Standard | Medical |
|  | Surgical Outcome Prediction | | Own data | Muller et al., 2018 | Not specified | Unsupervised | Soft clustering | n/a | Standard | Medical |
|  |  |  |  | Tomlinson et al., 2017 | Strips/grids | Standard | SVM |  | Higher order | Medical |
|  |  |  |  | Memarian et al., 2015 | Depth electrodes | Standard | LDA, NB, SVM |  | Higher order | Engineering |
|  | Pathological Tissue Detection | | Own data | Nejedly et al., 2019 | Not specified | Deep | LSTM | n/a | Standard | Multidisciplinary |
|  |  |  |  | Cimbalnik et al., 2019 | Strips/grids, Depth electrodes | Standard | SVM |  | Higher order | Medical |
|  |  |  |  | Nejedly et al., 2018 | Strips/grids | Deep | CNN |  | Standard | Medical |
|  | Bad Channel Detection | | Own data | Tuyisenge et al., 2018 | sEEG | Standard | Ensemble bagging model | n/a | Higher order | Medical |
|  | Tumour Tissue Detection | | Own data | Boussen et al., 2016 | Strips/grids | Standard | ANN | n/a | Standard | Medical |
| Motor | Movement Classification | | Own data | Pailla et al., 2019 | Strips/grids | Standard & Deep | DNN, LDA | n/a | Higher order | Engineering |
|  |  |  |  | Thomas et al., 2019 | Strips/grids | Standard | LDA |  | Standard | Engineering |
|  |  |  | BCI Competition IV | Xie et al., 2018 | Strips/grids | Deep | LSTM |  | Higher order | Engineering |
|  |  |  | Own data | Pan et al., 2018 | Strips/grids | Standard & Deep | LSTM, LR, SVM, MLP |  | Higher order | Medical |
|  |  |  |  | Combrisson et al., 2017 | sEEG | Standard | LDA, NB, KNN, SVM, RF |  | Standard | Medical |
|  |  |  |  | Combrisson et al., 2015 | sEEG | Standard | LDA |  | Standard | Medical |
|  |  |  |  | McMullen et al., 2013 | Strips/grids, Depth electrodes | Standard | LDA |  | Standard | Engineering |
|  |  |  |  | Benz et al., 2012 | Strips/grids | Standard | GRNN |  | Higher order | Engineering |
|  |  |  |  | Yanagisawa et al., 2009 | Strips/grids | Standard | SVM |  | Standard | Medical |
|  |  |  |  | Scherer et al., 2009 | Strips/grids | Standard | DSLVQ |  | Standard | Medical |
|  | Motor Imagery | | BCI Competition III | Rashid et al., 2020 | Strips/grids | Deep | LSTM | Training  99.64% (acc), 100% (ss), 99.28 (sp), 99.28% (prec)  Testing  97% (acc), 96% (ss), 98% (sp), 98% (prec) | Standard | Engineering |
|  |  |  |  | Li et al., 2015 | Strips/grids | Standard | LVQ | 92% (acc) | Higher order | Engineering |
|  |  |  |  | Yang et al., 2012 | Strips/grids | Standard | ANN | Training  88% (acc)  Validation  80% (acc)  Testing  80% (acc)  Overall  86% | Higher order | Medical |
|  |  |  |  | Demirer et al., 2009 | Strips/grids | Standard | SVM | Training  95%  Testing  73% | Higher order | Medical |
|  |  |  | Own data | Andrade et al., 2017 | Strips/grids | Standard | SVM | n/a | Standard | Medical |
|  |  |  |  | Hill et al., 2006 | Strips/grids | Standard | SVM |  | Higher order | Engineering |
|  | Speech Production | | Own data | Makin et al., 2020 | Strips/grids | Deep | LSTM | n/a | Higher order | Medical |
|  |  |  |  | Angrick et al., 2019 | Strips/grids | Deep | CNN |  | Standard | Medical |
|  |  |  |  | Angrick et al., 2019 | Strips/grids | Deep | CNN |  | Standard | Engineering |
|  |  |  |  | Livezey et al., 2019 | Strips/grids | Standard & Deep | LR, DNN |  | Standard | Multidisciplinary |
|  |  |  |  | Ramsey ^et^ al., 2018 | Strips/grids | Standard | SVM |  | Higher order | Medical |
|  |  |  |  | Ikeda et al., 2014 | Strips/grids | Standard | SVM |  | Standard | Medical |
| Cognitive Tasks | | | Own data | RaviPrakash et al., 2020 | Strips/grids | Deep | CNN, LSTM+CNN | n/a | Higher order | Medical |
|  |  |  |  | Saboo et al., 2019 | Strips/grids, depth electrodes | Unsupervised | GMM, SVM, LDA |  | Standard | Multidisciplinary |
|  |  |  |  | Weidemann et al., 2019 | Strips/grids, depth electrodes | Standard | LR |  | Standard | Medical |
|  |  |  |  | Hermiz et al., 2018 | Strips/grids, depth electrodes | Standard | LR |  | Standard | Medical |
|  |  |  |  | Derner et al., 2018 | Depth electrodes | Standard | SVM |  | Standard | Medical |
|  |  |  |  | Arora et al., 2018 | sEEG | Standard & Deep | LSTM, LR, SVM |  | Higher order | Engineering |
|  |  |  |  | Kragel et al., 2017 | Strips/grids, depth electrodes | Standard | LR |  | Standard | Medical |
|  |  |  |  | Schrouff et al., 2016 | Strips/grids | Standard | SVM |  | Standard | Medical |
| Sleep Staging | | | Own data | Kremen et al., 2019 | Strips/grids, depth electrodes | Unsupervised | BSC | n/a | Standard | Engineering |
|  |  |  |  | Rutigliano et al., 2018 | Not specified | Standard | ANN |  | Standard | Engineering |
|  |  |  |  | Kremen et al., 2017 | Depth electrodes | Standard | SVM |  | Standard | Engineering |
| Multiple | | HFO Detection / Classification  &  SOZ / Epileptic Focus Localization | Own data | Lai et al., 2020 | Strips/grids | Deep | CNN | n/a | Standard | Engineering |
|  |  | Seizure Detection / Prediction  &  Movement Classification | iEEG Portal | Zhu et al., 2020 | Strips/grids | Standard | DT |  | Standard | Engineering |
|  |  | Seizure Detection / Prediction  &  Sleep Staging | Own data | Principe et al., 2019 | sEEG | Standard | SVM |  | Higher order | Medical |
|  |  | Motor Classification  &  Motor Imagery | Own data | Shenoy et al., 2008 | Strips/grids | Standard | LDA, SVM, LPM, LSFD |  | Standard | Engineering |

**Keywords**

Performance measures

**acc** = accuracy

**auc** = area under curve

**fdr** = false detection rate

**fnr** = false negative rate

**fpr** = false positive rate

**NPV** = negative predictive value

**PPV** = positive predictive value

**prec** = precision

**sp** = specificity

**ss** = sensitivity

Algorithm

**BSC =** Behavioural State Classifier

**CNN =** Convolutional Neural Network

**DNN =** Deep Neural Network

**DNN =** Deep Neural Network

**DSLVQ =** Distinction-sensitive learning vector

**DT =** Decision Trees

**ELM =** Extreme Learning Machine

**EM =** Expectation-maximization

**ENN =** Elman Neural Network

**FCM =** Fuzzy-C-Means

**FCM-QEM-Based EM-GMM =** Fuzzy-C-Means Quantization Error Modeling Based Expectation-Maximization-Gaussian Mixture Model

**FFNN =** Feed Forward Neural Network

**GAN-NN =** Generative Adversarial Network Neural Network

**GCNN =** Graph Convolutional Neural Network

**GMM =** Gaussian Mixture Model

**GRNN =** General Regression Neural Network

**HD =** Hyperdimensional Computing

**JGRN =** Joint Graph Structure and Representation Learning Network

**KCRC =** Kernel Collaborative Representation Classifier

**KNN =** K Nearest Neighbours

**LDA =** Linear Discriminant Analysis

**LPM =** Linear Programming Machine

**LR =** Logistic Regression

**LSFD =** Linear Sparse Fisher’s Discriminant

**LVQ** **=** Learning Vector Quantization Neural Network

**MLP =** Multi-Layered Perceptron

**NB =** Naïve Bayes

**NNMF =** Non-Negative Matrix Factorization

**PFNN =** Particle Filter Neural Network

**PNN** = Probabilistic Neural Network

**QDA =** Quadratic Discriminant Analysis

**QEM =** Quantization Error Modeling

**RF =** Random Forest

**RVM =** Relevance vector machine

**SVM =** Support Vector Machine

**WNN =** Wavelet Neural Network

**XGB =** XGBoost
